# Protective effect of carboxymethylated chitosan on hydrogen peroxide-induced apoptosis in nucleus pulposus cells

**DOI:** 10.3892/mmr.2014.2942

**Published:** 2014-11-13

**Authors:** BIN HE, HAIYING TAO, SHIQING LIU, AILIN WEI

**Affiliations:** Department of Orthopaedics, Renmin Hospital of Wuhan University, Wuhan, Hubei 430060, P.R. China

**Keywords:** apoptosis, intervertebral disc degeneration, nucleus pulposus cells, carboxymethylated chitosan

## Abstract

Although the etiology of intervertebral disc degeneration is poorly understood, one approach to prevent this process may be to inhibit apoptosis. In the current study, the anti-apoptotic effects of carboxymethylated chitosan (CMCS) in nucleus pulposus (NP) cells were investigated with the aim to enhance disc cell survival. Rat NP cells were isolated and cultured *in vitro*, and hydrogen peroxide (H_2_O_2_) was used to build the NP cell apoptosis model. Cell viability was assessed with a cell counting kit-8 assay. The ratio of apoptotic cells was surveyed by annexin V-fluorescein isothiocyanate (FITC) and propidium iodide (PI) double staining analysis, and the morphology was observed by Hoechst 33342 staining. The mitochondrial membrane potential of NP cells was evaluated by rhodamine 123 fluorescence staining. Reverse transcription (RT)-quantitative polymerase chain reaction (qPCR) was performed to measure mRNA levels of inducible nitric oxide synthase (iNOS), caspase-3, B-cell lymphoma (Bcl)-2, type II collagen and aggrecan. Western blot analysis was performed to detect protein levels of iNOS and Bcl-2. The annexin V-FITC/PI and Hoechst 33342 staining results indicated that CMCS was able to prevent NP cells from apoptosis in a dose-dependent manner. Rhodamine 123 staining clarified that CMCS reduced the impairment of the mitochondrial membrane potential in H_2_O_2_-treated NP cells. Reduced caspase-3 and increased Bcl-2 activity were detected in CMCS-treated NP cells by RT-qPCR and western blot analysis. CMCS also promoted the proliferation and secretion of type II collagen and aggrecan in H_2_O_2_-treated NP cells. CMCS was indicated to be effective in preventing apoptotic cell death *in vitro*, demonstrating the potential advantages of this therapeutic approach in regulating disc degeneration.

## Introduction

Degeneration of the intervertebral disc (IVD) is a primary cause of lower back pain (LBP) and is a prerequisite for the occurrence of IVD hernia (1), which has a high social and economic cost. Regardless, the pathological mechanism of IVD degeneration remains to be fully defined. It is commonly accepted that IVD degeneration is influenced by numerous factors, including age, genetics and mechanical stimuli, of which the latter is the most important (2–6). Although mechanical stress is established to be an important modulator of degeneration, the underlying molecular mechanism of nucleus pulposus (NP) cells in the degeneration of the IVDs remains to be fully elucidated.

The IVD is composed of two conspicuous and interdependent anatomical structures: The surrounding annulus fibrosus (AF) and the central gelatinous NP. IVD cells, particularly NP cells, are crucial to maintain the integrity of the IVD, which occurs through producing type II collagen, aggrecan and other components involved in extracellular matrix (ECM) metabolism. A reduced NP cell population and loss of ECM are central features in the aging and degeneration of IVDs. Previous evidence has suggested that the NP may be associated with aging and the initiation of IVD degeneration (7–9). One study, focusing on the cellular mechanobiology of IVD, suggested that NP cells possess distinct characteristics necessary for IVD homeostasis (10). Although the precise molecular mechanism of IVD degeneration remains unclear, previous studies have suggested that the apoptosis or programmed cell death in NP cells may be one of the key factors (11,12).

Apoptosis is an active mode of cell death that is observed in healthy cells and tumor cells, in physiological and pathological situations, and is distinct from passive cell death (necrosis) (13). The signaling events leading to apoptosis can be divided into two distinct pathways, either involving the mitochondria or death receptors. In the mitochondrial pathway, death signals lead to changes in mitochondrial membrane permeability, and the subsequent release of pro-apoptotic factors, such as cytochrome *c* from the mitochondria. Once in the cytoplasm, cytochrome *c* catalyzes the oligomerization of apoptotic protease activating factor-1 (Apaf-1) (14). This promotes the activation of procaspase-9, which then initiates a caspase cascade involving the downstream executioner, procaspase-3, which in turn activates a DNase, termed caspase-activated DNase (15,16). In the death receptor pathway, apoptosis is triggered by cell surface death receptors, including Fas and the tumor necrosis factor (TNF) receptor, which contain death domains. These death domains recruit adaptors and induce the activation of initiator caspase-8, followed by cleavage of downstream effector caspase and various substrates. Park *et al* (17) established that NP cells participate in the intrinsic pathway and subsequently undergo apoptotic cell death through mitochondrial involvement. The cellular commitment to apoptosis is regulated by the B-cell lymphoma (Bcl)-2 family of proteins, which consists of apoptosis agonists (Bax, Bak and Bad) and antagonists (Bcl-2 and Bcl-xl). The balance between pro-apoptotic proteins, such as Bax, and anti-apoptotic proteins, such as Bcl-2, is considered to be a crucial factor in the regulation of apoptosis. Bax and Bcl-2 are mitochondrial proteins, and have been demonstrated to be associated with the regulation of mitochondrial membrane permeability. Bax exerts its pro-apoptotic activity by translocating from the cytoplasm to the mitochondria, and inducing cytochrome *c* release from isolated mitochondria. However, Bcl-2 exerts its anti-apoptotic activity, at least in part, by inhibiting the translocation of Bax to the mitochondria.

Carboxymethylated chitosan (CMCS) is a soluble derivative of chitosan and it possesses numerous desirable physiochemical and biological features. It has been indicated previously that CMCS can significantly suppress the degeneration of cartilage in osteoarthritis and protect chondrocytes from interleukin-1β-induced catabolism and apoptosis (18,19). It has been previously observed that CMCS can stimulate proliferation and the secretion of NGF in cultured Schwann cells (SCs) by activation of the mitogen-activated protein kinase/extracellular signal-regulated kinase, phosphatidylinositide 3-kinase/Akt and Wnt/β-catenin signaling cascades (20,21). The protection of NP cells from apoptosis possesses great potential for the treatment of IVD degeneration, and the present study aims to determine whether CMCS serves a similar function in NP cells as in chondrocytes and SCs.

The aim of the current study was to investigate whether CMCS is effective in preventing hydrogen peroxide (H_2_O_2_)-induced apoptotic cell death, and to discuss the potential advantages of this approach in providing a therapeutic approach to the regulation of IVD degeneration.

## Materials and methods

### Animals and reagents

24 healthy male Sprague-Dawley (SD) rats with an average body weight (BW) of 362±35 g were selected as NP cell donors (obtained from the Center of Experimental Animals of Wuhan University, Wuhan, China). Dulbecco’s modified Eagle’s medium/Ham’s F-12 (DMEM/F-12) was obtained from Gibco Life Technologies (Carlsbad, CA, USA) and fetal bovine serum (FBS) was obtained from HyClone (Logan, UT, USA). Carboxymethylated chitosan (CMCS, purity >99%) was supplied by the Institute of Chemistry and Environmental Science of Wuhan University. A cell counting kit-8 (CCK-8) was purchased from Dojindo Molecular Technologies, Inc. (Kumamoto, Japan). Primers were provided by Invitrogen Life Technologies (Carlsbad, CA, USA). Rabbit polyclonal anti-Bcl-2 (#2876) and rabbit monoclonal anti-β-actin (13E5; #4970) antibodies were obtained from Cell Signaling Technology, Inc. (Beverly, MA, USA). The anti-inducible nitric oxide synthase rabbit polyclonal (iNOS; sc-651) antibody was from Santa Cruz Biotechnology, Inc. (Dallas, TX, USA). Rhodamine 123 (Rho123) and Hoechst 33342 were obtained from Sigma-Aldrich (St. Louis, MO, USA). Phosphate-buffered saline (PBS, ×10, ST476) and SDS-PAGE Gel Kit (P0012A), were obtained from the Beyotime Institute of Biotechnology (Haimen, China) and were of the highest purity commercially available.

### Cell isolation and culture

5 SD rats (aged 10–12 weeks, weighing 362±35 g) were enrolled in the present study. Rat NP cells were isolated using a previously described explant culture method (22). Briefly, rats were euthanized with an overdose of intravenous pentobarbital (100 mg/kg body weight; Shanghai Biorui Biological Technological Co., Ltd., Shanghai, China), and the lumbar IVDs were resected from the spinal column. The gel-like NP tissue was separated from the AF using a dissection microscope (Five-Lake Medical Devices Co., Ltd., Wuhan, China) under aseptic conditions. The gelatinous NP tissues obtained from each animal were cut into small pieces (<1 mm^3^) immediately, then digested with 0.1% type-2 collagenase (Sigma-Aldrich) in DMEM/F-12 at 37°C in a KYC-100C gyratory shaker from Shanghai Fuma Laboratory Instrument Company (Shanghai, China) at 110 rpm. After 4 h, the suspension was filtered through a 70-μm mesh. The filtered cells were washed with DMEM/F-12 and then seeded into 25 cm^2^ culture flasks. The cells were incubated in DMEM/F-12 with 10% FBS and a penicillin-streptomycin solution (SV30010; HyClone; 100 U/ml streptomycin and 100 U/ml penicillin) in a 5% CO_2_ incubator. The medium was refreshed every 3 days. The NP cells were chondrocyte-like cells, identified by type II collagen and aggrecan immunohistostaining.

### Establishment of apoptotic models of NP cells

To establish the apoptotic model of cultured NP cells, H_2_O_2_ (Wuhan Boster Biological Technology Company, Wuhan, China). was used as described previously (23). Briefly, NP cells (cell density of 1×10^6^/ml) were cultured overnight at 37°C in the culture medium as described above. Different concentrations of H_2_O_2_ (100, 200 and 300 μM) were used to induce the damage to NP cells. NP cells were examined at 6, 12 and 24 h subsequent to the addition of H_2_O_2_. To determine the effects of CMCS on H_2_O_2_-induced apoptosis in NP cells, cell cultures were treated with H_2_O_2_ for 6 h and then the culture medium was replaced immediately by fresh medium with CMCS. The concentrations of CMCS were 50, 100 and 200 μg/ml.

### Cell viability assay

Cell viability was assessed by CCK-8 assay. Cells were suspended at a final concentration of 2×10^4^ cells/well and cultured in 96-well flatbottomed microplates with the DMEM/F-12 containing 0.1% FBS. The medium was replaced 24 h later with DMEM/F-12 containing H_2_O_2_, CMCS or phosphate-buffered saline (PBS; control group). For assessing the cytotoxic effect of H_2_O_2_ with CMCS, cells were incubated with 100, 200 and 300 μM H_2_O_2_ without CMCS, and 300 μM H_2_O_2_ with different concentrations of CMCS (50, 100 and 200 μg/ml) for the indicated time intervals. For quantitative analysis of the cell proliferation, 10 μl CCK-8 solution was added to each well of a 96-well flat bottomed microplate containing 100 μl DMEM/F-12, and the plate was incubated at 37°C for 1 h in a 5% CO_2_ atmosphere. The optical density, which is proportional to cell metabolic activity, was measured at 450 nm using an ELx800 Absorbance Microplate Reader (BioTek Instruments, Inc., Winooski, VT, USA). Cell viability was expressed as a percentage of the number of control (untreated) cells. Viability in the control group was designated as 100%. The cell viability of each group was calculated as follows: Cell viability (% of control) = [(Ae−Ab)/(Ac−Ab)] × 100. Ae, Ab and Ac represent the A450 of the experimental, blank and control groups, respectively. All experiments were performed in triplicate in three independent experiments.

### Annexin V-fluorescein isothiocyanate(FITC)/*propidium iodide* (PI) staining

The level of apoptotic death in the NP cells was determined using flow cytometric analysis. Cellular apoptosis was observed by annexin V-FITC/PI double staining, performed using an Annexin V/FITC Apoptosis Detection kit I (no. 556547; BD Biosciences, Franklin Lakes, NJ, USA) according to the manufacturer’s instructions. Briefly, cells were cultured at a density of 6×10^5^ cells/ml and seeded in 6-well plates. The cells were cultured in DMEM/F-12 containing various concentrations of H_2_O_2_ (100, 200 and 300 μM) for 6, 12 or 24 h, or CMCS at various concentrations (50, 100 and 200 μg/ml) for 3 h followed by the addition of H_2_O_2_ for 24 h for the indicated time. Cells were harvested by trypsinization (Gibco-BRL, Rockville, MD, USA), then washed twice with cold PBS and centrifuged at 400 × g. Approximately 1×10^5^–1×10^6^ cells were then suspended in 500 μl binding buffer from the apoptosis detection kit, centrifuged again at 400 × g for 5 min and then the supernatant was removed. Cells were resuspended in 500 μl binding buffer and transferred to a sterile flow cytometry glass tube. Annexin V-FITC (5 μl) and PI (5 μl) were added prior to incubation in the dark at room temperature. Cells were analyzed with a flow cytometer (BD Biosciences) at 488 nm. The distribution of cells was analyzed using Cell Quest Pro software (version 4.01; BD Biosciences) in the BD FACSVerse™ flow cytometer (BD Biosciences) within 1 h of staining. Data from 10,000 cells were collected for each data file. Apoptotic cells were identified as the annexin V-FITC-positive and PI-negative cells. Finally, the number of cells in each category was expressed as a percentage of the total number of stained cells.

### Nuclear staining with Hoechst 33342

Apoptotic nuclear morphology was assessed with Hoechst 33342 (Sigma-Aldrich) staining. To determine whether CMCS protects from recognized morphological features of apoptosis, such as H_2_O_2_-induced chromatin condensation and fragmentation, cells were cultured in 6-well plates (3.0×10^5^ cells/well) with DMEM/F-12 containing 10% FBS, then treated for 24 h with 300 μM H_2_O_2_ and CMCS at 37°C in a humidified atmosphere of 5% CO_2_. Cell apoptosis was evaluated by Hoechst 33342 staining as described previously (24). Briefly, following 24 h culture in the DMEM/F-12 medium, the cells were stained with 10 μg/ml Hoechst 33342 at 37°C for 20 min. The cells were washed and suspended again in PBS for morphological observation under a IX51 fluorescence microscope (Olympus Corporation, Tokyo, Japan) with excitation at 355 nm and emission at 465 nm. A minimum of 400 cells from six randomly selected fields per dish were counted, and each treatment was performed in triplicate.

### Measurement of mitochondrial membrane potential (ΔΨm)

Changes in ΔΨm were estimated by the uptake of Rho123, a cell-permeant, lipophilic, cationic, fluorescent dye that permeates easily and interacts with negative charges on the inner mitochondrial membrane at a low concentration. It accumulates in normal mitochondria, but a decline in ΔΨm leads to leakage of Rho123 from the mitochondria, thus the fluorescence intensity is reduced. Therefore, the effects of H_2_O_2_, CMCS and a combination of the two on ΔΨm were assessed as one of the markers of mitochondrial function. Briefly, H_2_O_2_ or H_2_O_2_/CMCS treatments were performed for 24 h. At the end of incubation, treated NP cells were incubated with Rho123 (10 μg/ml) at 37°C for 20 min. Subsequently, they were washed twice with PBS and then observed with the excitation filter set at 488 nm and the emission filter at 510 nm under the fluorescence microscope.

### Reverse transcription (RT)-quantitative polymerase chain reaction (qPCR)

Total RNA was extracted using TRIzol reagent (Invitrogen Life Technologies, Carlsbad, CA, USA) according to the manufacturer’s protocol. The RNA samples were quantified by spectrophotometry at 260 and 280 nm (A260/A280 ~2.0; A260 = 40 μg RNA/ml) by the NanoDrop (ND-8000) Spectrophotometer (Thermo Fisher Scientific, Braunschweig, Germany). RNA was then reverse-transcribed to cDNA using a Reverse Transcription-Polymerase Chain Reaction kit (Takara Biotechnology Co., Ltd., Dalian, China) according to the manufacturer’s instructions. The cDNA was analyzed immediately or stored at −20°C. qPCR amplification was performed with an ABI Prism 7900HT Real-Time PCR system (Applied Biosystems Life Technologies, Foster City, CA, USA), and the SYBR Green I fluorescent dye method was used to quantify cDNA. PCR cycling conditions consisted of an initial denaturing step for 10 sec at 95°C; then 40 cycles of 5 sec each at 95°C; followed by 30 sec at 60°C. A stable and reliable standard curve was established by plotting the threshold cycle (Ct) values. Following amplification, a melting curve analysis was performed in order to verify the authenticity of the amplified product by its specific melting temperature (Tm). GAPDH was used as the internal control. The relative levels of mRNA of the target genes were then calculated, through which the gene expression level and the trend of change were determined. The specificity of each reaction was controlled by melting curve analysis. A negative PCR control containing water in place of cDNA was prepared. The relative levels of mRNA were analyzed by the 2^−ΔΔCt^ method. qPCR was conducted in triplicate in three independent experiments. The sequences of the primers are presented in Table I.

### Western blot analysis

NP cells were treated with H_2_O_2_ in the presence or absence of CMCS. Samples of the cell cultures were treated with lysis buffer (P0013, Beyotime Institute of Biotechnology) containing 150 mM NaCl, 10 mM Tris-HCl, 1 mM EDTA, 1% Triton X-100, 10% glycerol, 1 mM phenylmethylsulphonyl fluoride, 10 μg/ml leupeptin and 10 μg/ml aprotinin. Lysates were subsequently centrifuged at 13,000 × g for 15 min and the supernatant was collected for protein analysis. Sample protein concentration was determined using a commercial bicinchoninic acid protein assay kit (Pierce Biotechnology, Inc., Rockford, IL, USA). Equal amounts of protein from cell lysates were resuspended in sample buffer (P0015; Beyotime Institute of Biotechnology) containing 62 mM Tris-HCl (pH 6.8), 2% sodium dodecylsulphate (SDS), 10% glycerol, 5% β-mercaptoethanol and 0.04% bromphenol blue, then resolved by SDS-PAGE and transferred to polyvinylidene difluoride membranes (EMD Millipore, Billierica, MA, USA). Following brief washing in Tris-buffered saline with Tween-20 (TBST) [25 mM Tris-HCl (pH 7.5), 50 mM NaCl, 0.1% Tween-20; Beijing Biosntech Co., Beijing, China], the membrane was blocked with 5% (w/v) non-fat dried milk in TBST overnight at 4°C. The membrane was incubated for 3 h with the appropriate primary antibodies. Following washing with TBST, the membranes were incubated with the respective goat anti-rabbit peroxidase-conjugated secondary (#7074; Cell Signaling Technology, Inc.) antibodies for 1 h then washed again with TBST. Immunodetection was accomplished by enhanced chemiluminescence using an enhanced chemiluminescence detection kit for HRP (Pierce Biotechnology, Inc.) followed by autoradiography on Kodak-X-OMAT-AR film (Kodak, Rochester, NY, USA) and performed using a Geliance 200 Gel Imaging system (PerkinElmer, Inc., Rocky Hill, NJ, USA) and GeneSnap software, version 6.08.04 (Syngene, Frederick, MD, USA). Bands were analyzed using the GeneTools software, version 3.07.04 (Syngene). All data are expressed as the relative differences between control and treated cells, subsequent to normalization to the β-actin expression level.

### Statistical analysis

Data are presented as the mean ± standard error. Differences between groups were compared using one-way analysis of variance on SPSS, version 16.0 (SPSS, Inc., Chicago, IL, USA). P<0.05 was considered to indicate a statistically significant difference.

## Results

### Effect of CMCS on cell viability in H_2_O_2_-treated NP cells

**Figure 1:**
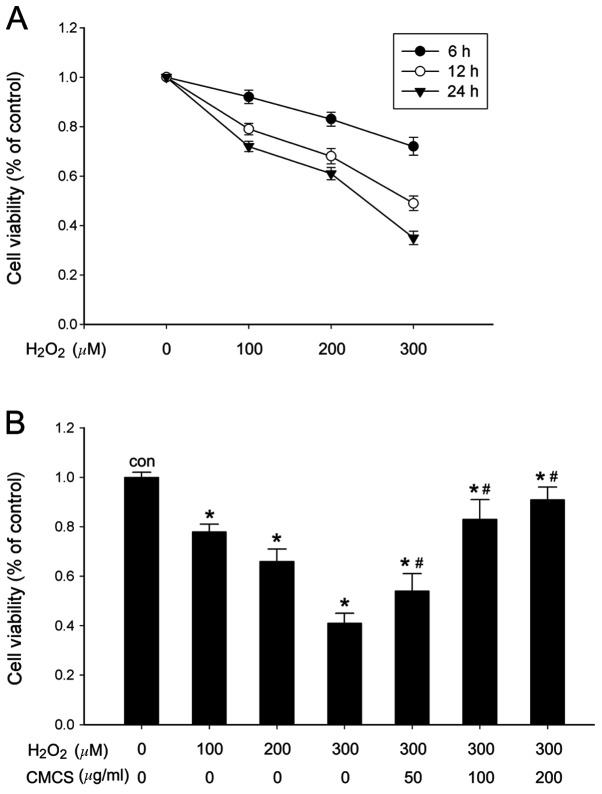
Effect of CMCS on cell viability in H_2_O_2_-induced NP cells as assessed by CCK-8 assay. (A) Time and dose-dependent manner of the effect of H_2_O_2_ on cell viability in NP cells. (B) Effect of CMCS on cell viability in H_2_O_2_-treated NP cells. Cells were treated with H_2_O_2_ for 24 h with or without 3-h pretreatment with CMCS. ^*^P<0.05 vs. the control group; ^#^P<0.05 vs. the 300 μM H_2_O_2_-induced NP cells group. CMCS, carboxymethylated chitosan; H_2_O_2_, hydrogen peroxide; NP, nucleus pulposus; CCK, cell counting kit.

The NP cell viability and metabolic activity were analyzed by CCK-8 assay. The results indicated that different concentrations of H_2_O_2_ stimulation (100, 200 and 300 μM) were able to reduce the number of metabolically active cells and viability in a time- and dose-dependent manner (Fig. 1A). A significant reduction in cell viability was observed at 24 h following 300 μM H_2_O_2_ exposure (Fig. 1B). However, when NP cells were pretreated with CMCS (50, 100 or 200 μg/ml) for 3 h and then exposed to H_2_O_2_ for 24 h, cell viability was improved in a dose-dependent manner. The most significant increase was observed in the 200 μg/ml CMCS-treated group compared with cell viability following 300 μM H_2_O_2_ treatment (Fig. 1B).

### Effect of CMCS on apoptosis in H_2_O_2_-treated NP cells

**Figure 2:**
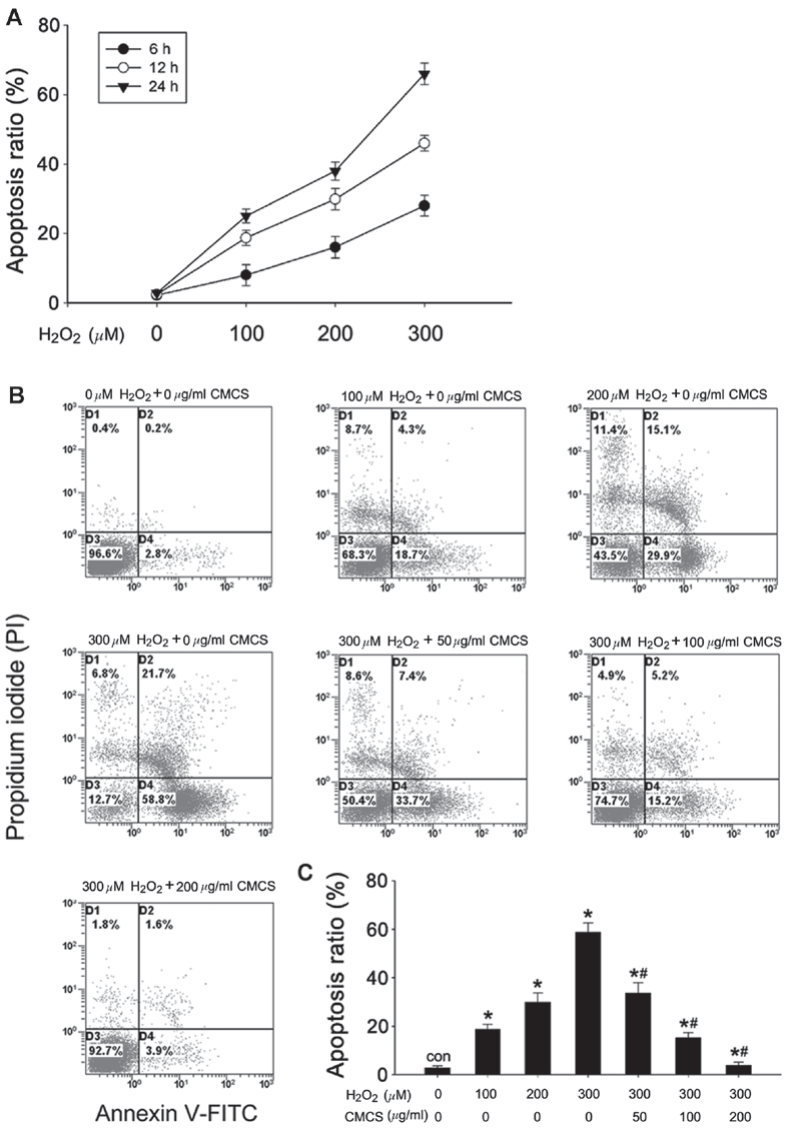
Effect of CMCS on apoptosis in H_2_O_2_-treated NP cells. (A) Time- and dose-dependent manner of the effect of H_2_O_2_ on apoptotic rates in NP cells. (B) Percentage distribution graphs displaying the effect of CMCS on apoptotic rates in H_2_O_2_-treated NP cells. (C) HIstogram summarizing percentages of apoptotic cells in each group following 24-h H_2_O_2_ exposure with or without 3-h CMCS pretreatment. ^*^P<0.05 vs. the control group; ^#^P<0.05 vs. the 300 μM H_2_O_2_-induced group. CMCS, carboxymethylated chitosan; H_2_O_2_, hydrogen peroxide; NP, nucleus pulposus; V-FITC, V-fluorescein isothiocyanate.

The rate of apoptosis was quantified using flow cytometry with annexin V-FITC/PI staining. As presented in Fig. 2A, H_2_O_2_ exposure increased the apoptotic rates of the NP cells compared with the control cells, in a time- and dose-dependent manner. The apoptotic ratios were 18.7, 29.9 and 58.8% in 100, 200 and 300 μM H_2_O_2_-treated NP cells respectively, while it was 2.8% in control cells (Fig. 2B and C). CMCS significantly inhibited H_2_O_2_-induced apoptosis in a dose-dependent manner; treatment with 50, 100 and 200 μg/ml CMCS in H_2_O_2_-treated NP cells resulted in apoptotic ratios of 33.7, 15.2 and 3.9%, respectively.

### Effect of CMCS on nucleic morphology in H_2_O_2_-treated NP cells

**Figure 3:**
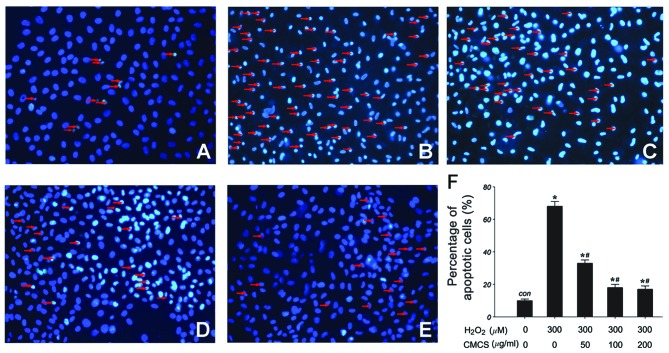
Effect of CMCS on nucleic morphology of H_2_O_2_-treated NP cells. NP cells were incubated with H_2_O_2_ with or without CMCS pretreatment then stained with Hoechst 33342 to detect apoptotic morphology. (A) Control untreated group; (B) 300 μM H_2_O_2_; (C) 300 μM H_2_O_2_+50 μg/ml CMCS; (D) 300 μM H_2_O_2_+100 μg/ml CMCS; and (E) 300 μM H_2_O_2_+200 μg/ml CMCS. Red arrows represent cells with DNA condensation. (F) The numbers of cells with broken DNA per 30 cells in three different fields in each condition of one experiment were counted and repeated for three different experiments. The mean percentages of positive staining are presented as a histogram. ^*^P<0.05 vs. the control group; ^#^P<0.05 vs. the 300 μM H_2_O_2_-induced group. CMCS, carboxymethylated chitosan; H_2_O_2_, hydrogen peroxide; NP, nucleus pulposus.

Subsequent to culture with H_2_O_2_ or the H_2_O_2_/CMCS combination, morphological changes in the NP cells were observed by Hoechst 33342 staining. As presented in Fig. 3, in the control group, NP cell nuclei were round and stained homogeneously with Hoechst 33342 (Fig. 3A). In H_2_O_2_-treated NP cells, a considerable proportion of cells displayed characteristics of apoptosis with condensed and fragmented nuclei (Fig. 3B). Treatment with 50, 100 and 200 μg/ml CMCS led to a significant reduction in the number of apoptotic cells with fragmented nuclei (Fig. 3C–F). These results suggest that CMCS is able to inhibit the H_2_O_2_-induced nucleic morphological changes in NP cells.

### Effects of CMCS on ΔΨm in H_2_O_2_-treated NP cells

**Figure 4:**
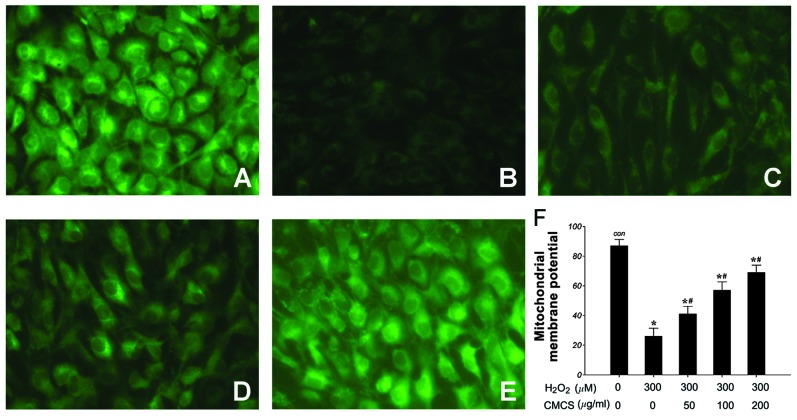
Effect of CMCS on mitochondrial membrane potential (ΔΨm) in H_2_O_2_-exposed NP cells. NP cells were incubated with H_2_O_2_ or H_2_O_2_/CMCS treatment and then stained with Rho123 to detect the ΔΨm. (A) Control untreated group; (B) 300 μM H_2_O_2_; (C) 300 μM H_2_O_2_+50 μg/ml CMCS; (D) 300 μM H_2_O_2_+100 μg/ml CMCS; and (E) 300 μM H_2_O_2_+200 μg/ml CMCS. (F) The ΔΨm was assessed by Rho123 fluorescence intensity. ^*^P<0.05 vs. the control group; ^#^P<0.05 vs. the 300 μM H_2_O_2_-induced group. CMCS, carboxymethylated chitosan; H_2_O_2_, hydrogen peroxide; NP, nucleus pulposus; Rho123, rhodamine123.

It has been reported that CMCS may prevent mitochondrial oxidative stress. Thus, the effects of H_2_O_2_ and the H_2_O_2_/CMCS combination on ΔΨm were examined as a marker of mitochondrial function. ΔΨm was assessed using the Rho123 fluorescent dye, the intensity of which reflects mitochondrial function. As demonstrated in Fig. 4, 300 μM H_2_O_2_ induced a significant reduction in ΔΨm following treatment for 24 h compared with the control group (Fig. 4A and B). Treatment with CMCS was demonstrated to prevent this reduction in a dose-dependent manner (Fig. 4C–F). These results suggest that CMCS may be able to protect mitochondrial function in NP cells.

### Effect of CMCS on the expression level of iNOS in H_2_O_2_-treated NP cells

**Figure 5:**
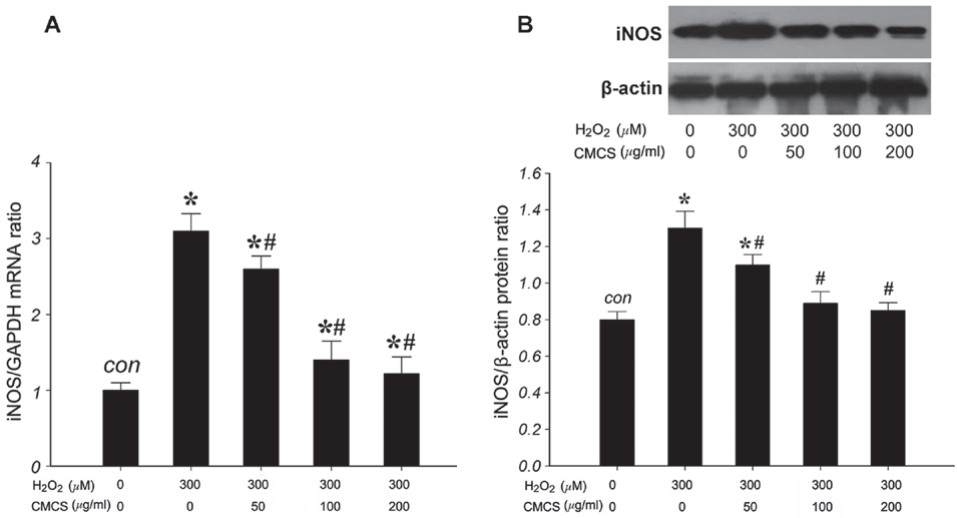
Effects of CMCS on iNOS expression in H_2_O_2_-treated NP cells. (A) RT-qPCR analysis of iNOS mRNA expression following H_2_O_2_-induced apoptosis with or without CMCS pretreatment. The data were normalized to GAPDH expression and presented as a percentage of the level in untreated cellular controls. (B) Top: Western blot analysis of iNOS protein expression following H_2_O_2_-induced apoptosis with or without CMCS pretreatment before apoptotic induction. Bottom: Quantitative analysis of the representative western blot. Data were normalized to β-actin expression and presented as a percentage of the level in untreated cellular controls. ^*^P<0.05 vs. the control group; ^#^P<0.05 vs. the 300 μM H_2_O_2_-induced group. CMCS, carboxymethylated chitosan; iNOS, inducible oxide synthase; H_2_O_2_, hydrogen peroxide; NP, nucleus pulposus; RT-qPCR, reverse transcription quantitative polymerase chain reaction.

To investigate the effects of CMCS on the expression of iNOS in H_2_O_2_-treated NP cells, the mRNA and protein levels of iNOS were measured. RT-qPCR results indicated that 300 μM H_2_O_2_ significantly increased the iNOS/GAPDH mRNA ratio compared with that of the control group (Fig. 5A). However, treatment with 50, 100 and 200 μg/ml CMCS was able to inhibit this increase in a dose-dependent manner. The expression of iNOS protein (130 kDa) was detected by western blot analysis (Fig. 5B). H_2_O_2_ exposure significantly increased the iNOS protein level compared with that of the control group, and treatment with 50, 100 and 200 μg/ml CMCS was able to inhibit this increase in a dose-dependent manner. These results suggest that CMCS is able to inhibit the H_2_O_2_-induced increase in NP cell iNOS mRNA and protein levels.

### Effect of CMCS on caspase-3 mRNA expression in H_2_O_2_-treated NP cells

**Figure 6:**
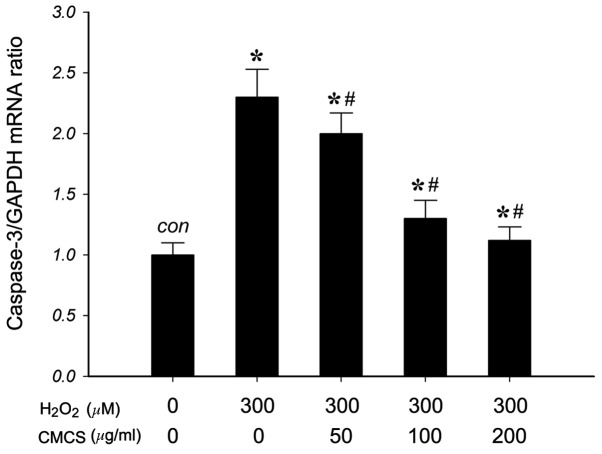
Effect of CMCS on caspase-3 expression level in H_2_O_2_-induced NP cells. RT-qPCR analysis of caspase-3 mRNA expression following H_2_O_2_ exposure with or without CMCS pretreatment. Data were normalized to GAPDH expression and presented as a percentage of the level in untreated cellular controls. ^*^P<0.05 vs. the control group; ^#^P<0.05 vs. the 300 μM H_2_O_2_-induced group. CMCS, carboxymethylated chitosan; H_2_O_2_, hydrogen peroxide; NP, nucleus pulposus; RT-qPCR, reverse transcription quantitative polymerase chain reaction.

To investigate the effects of CMCS on the expression of caspase-3 (a mediator of apoptosis) in H_2_O_2_-treated NP cells, the mRNA levels of caspase-3 were measured. As presented in Fig. 6, RT-qPCR results indicated that 300 μM H_2_O_2_ exposure significantly increased the level of caspase-3 mRNA compared with that of the control group. Treatment with 50, 100 and 200 μg/ml CMCS was able to inhibit this increase in a dose-dependent manner. These results suggested that CMCS can inhibit the H_2_O_2_-induced increase in the level of caspase-3 in NP cells.

### Effect of CMCS on the expression levels of Bcl-2 in H_2_O_2_-treated NP cells

**Figure 7:**
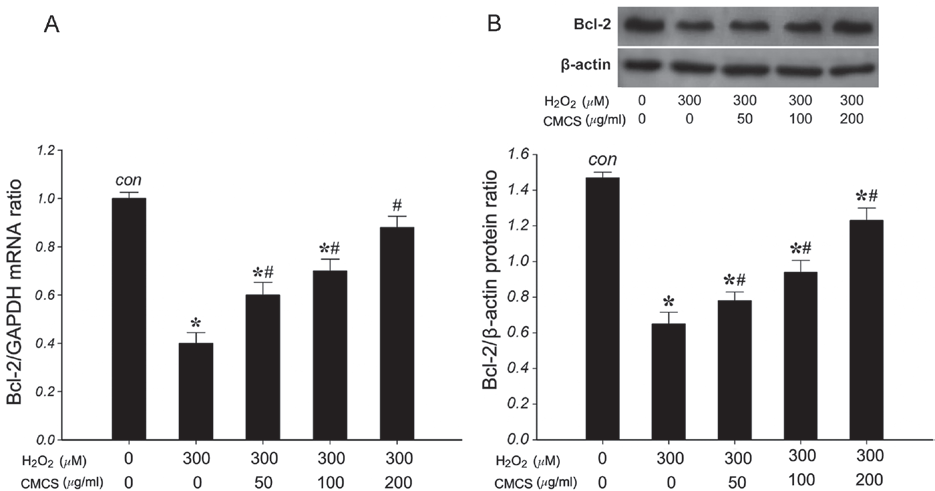
Effects of CMCS on Bcl-2 expression in H_2_O_2_-treated NP cells. (A) RT-qPCR analysis of Bcl-2 mRNA expression levels following H_2_O_2_ exposure with or without CMCS pretreatment. The data were normalized to GAPDH expression and presented as a percentage of the level in untreated cellular controls. (B) Top: Western blot analysis of Bcl-2 protein expression following H_2_O_2_-induced apoptosis with or without CMCS pretreatment. Bottom: Densitometry analysis of the representative western blot. Data were normalized to β-actin expression and presented as a percentage of untreated cellular controls. ^*^P<0.05 vs. the control group; ^#^P<0.05 vs. the 300 μM H_2_O_2_-treated group. CMCS, carboxymethylated chitosan; Bcl, B-cell lymphoma; H_2_O_2_, hydrogen peroxide; NP, nucleus pulposus; RT, reverse transcription; qPCR, quantitative polymerase chain reaction.

To investigate the effects of CMCS on the expression of Bcl-2 in H_2_O_2_-treated NP cells, the mRNA and protein levels of Bcl-2 were measured. RT-qPCR results indicated that 300 μM H_2_O_2_ significantly reduced the level of Bcl-2 mRNA compared with that of the control group (Fig. 7A). However, treatment with 50, 100 and 200 μg/ml CMCS was able to inhibit this reduction in a dose-dependent manner. The expression of Bcl-2 protein (26 kDa) was detected by western blot analysis. As presented in Fig. 7B, 300 μM H_2_O_2_ significantly reduced the level of Bcl-2 protein compared with that in the control group. However, treatment with 50, 100 and 200 μg/ml CMCS was able to inhibit the reduction in Bcl-2 protein in H_2_O_2_-treated NP cells in a dose-dependent manner. These results suggest that CMCS is able to inhibit the H_2_O_2_-induced reduction of Bcl-2 mRNA and protein in NP cells.

### CMCS increases ECM production in H_2_O_2_-treated NP cells

**Figure 8:**
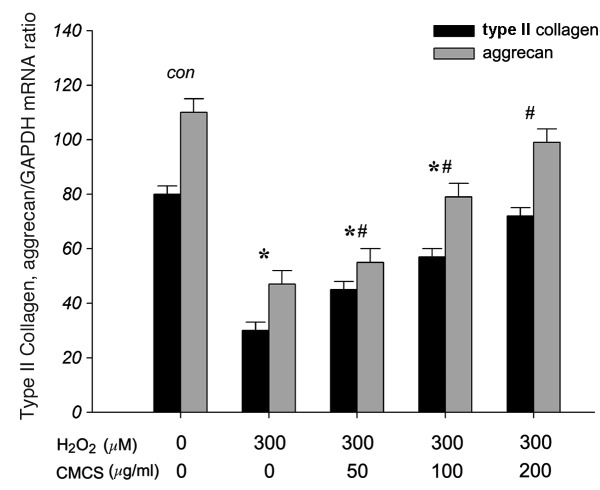
Effects of CMCS on ECM secretion in H_2_O_2_-induced NP cells. The RT-qPCR of type II collagen and aggrecan mRNA expression following H_2_O_2_ exposure with or without CMCS pretreatment. Data were normalized to GAPDH expression and presented as a percentage of the level in untreated cellular controls. ^*^P<0.05 vs. the control group; ^#^P<0.05 vs. the 300 μM H_2_O_2_-treated group. CMCS, carboxymethylated chitosan; ECM, extracellular matrix; H_2_O_2_, hydrogen peroxide; NP, nucleus pulposus; RT, reverse transcription; qPCR, quantitative polymerase chain reaction.

To investigate the effects of CMCS on the secretion of ECM components, including collagen type II and aggrecan, in H_2_O_2_-exposed NP cells, the mRNA levels of type II collagen and aggrecan were measured. As presented in Fig. 8, RT-qPCR results indicated that 300 μM H_2_O_2_ significantly reduced the levels of type II collagen and aggrecan mRNA compared with those of the control group. However, treatment with 50, 100 and 200 μg/ml CMCS was able to inhibit this reduction in a dose-dependent manner. These results suggest that CMCS is able to protect the secretion of type II collagen and aggrecan in the apoptotic environment.

## Discussion

The present study demonstrated that CMCS can protect or rescue NP cells *in vitro* from undergoing apoptosis following H_2_O_2_ exposure, and that the mechanisms of this protection may involve caspase-3 and Bcl-2 activation and mitochondrial function.

IVD degeneration is considered to be associated with genetic factors, in addition to excessive mechanical loading, which together alter the biomechanical properties of the IVD. Although the precise mechanism of disc degeneration remains unclear, it has been suggested in previous studies that apoptosis or programmed cell death of IVD cells may be one of the key steps in disc degeneration (25).

In the present study, it was observed that treatment of the NP cells with CMCS prior to exposure to H_2_O_2_ resulted in significantly increased cell survival. This was accompanied by the finding that CMCS, prophylactively added to the NP cell cultures, demonstrated a protective effect on the NP cells regarding the H_2_O_2_-induced reduction in viability. These results were consistent with previous observations that CMCS and chitosan were able to protect chondrocytes, endometriotic cells, vein endothelial cells and astrocytes from apoptosis (19,26–28).

There are numerous factors involved in apoptotic cascades. Caspases, the ‘key executioners’ of apoptosis, are a family of cysteine proteases capable of cleaving essential cellular substrates with aspartate residues (29). Caspases-8, -9 and -10 are involved in the initiation and amplification of apoptosis, while caspases-3, -6 and -7 are involved in executing the apoptotic program and cell death (30). Caspase-3, the most prominent effective caspase, is localized downstream in the caspase cascade and represents the main effective molecule in apoptosis. It irreversibly executes programmed cell death. Caspase-3 is located in the cellular cytoplasm in its inactive form in the normal microenvironment, but it is auto-proteolytically cleaved into an active form of the enzyme under apoptotic conditions (31,32). Apoptosis is triggered by several stimuli resulting in several apoptotic pathways. Rannou *et al* (33) demonstrated that mechanical overload induces disc degeneration via a caspase-9-dependent apoptotic pathway, suggesting that disc cell apoptosis is the primary cause of disc degeneration. Others have indicated that caspase-3 acts as the main apoptosis effector, and it may be the therapeutic target for regulation of IVD degeneration (34). In the present study, H_2_O_2_-exposed NP cells exhibited an increased level of intracellular caspase-3 mRNA and caspase-3 activity. Treatment with CMCS significantly inhibited the caspase-3 activity generated by H_2_O_2_. The results of the current study suggest that CMCS inhibits caspase-3 activity and the anti-apoptotic effect of CMCS is at least partly mediated via caspase-3 enzymatic inhibition.

There are two major signaling pathways controlling the initiation of apoptosis in mammals. The extrinsic pathway involves engagement of cell-surface death receptors by ligands that belong to the TNF receptor superfamily and the consequent activation of caspase-8. The intrinsic pathway involves caspase-9 as the initiator, and originates from the mitochondria. Stressed mitochondria release a set of molecules, including cytochrome-*c* and Apaf-1, to form the apoptosome molecular cluster that activates caspase-9 and its downstream effector, caspase-3. Park *et al* (17) examined human herniated lumbar disc tissues with the use of immunohistochemical staining and western blot analysis to determine the presence of several proteins associated with apoptosis. They established that the proteins associated with the intrinsic pathway were stained positive in all samples. The results of their study suggest that disc cells participate in the intrinsic pathway, and subsequently undergo apoptotic cell death through mitochondrial involvement.

In the intrinsic pathway, Bcl-2 prevents or delays apoptotic induction by a large variety of stimuli in various cell types (35). Molecular intervention at the level of Bcl-2 in the apoptotic pathway, therefore, has the potential to enhance cell survival. Although the apoptotic cascade remains to be fully elucidated, overexpression of Bcl-2 has previously been demonstrated to prevent the release of apoptotic induction factors and the subsequent activation of caspase-3 (36). Sudo and Minami (37) indicated that Bcl-2 overexpression in IVD cells effectively prevented *in vitro* apoptotic cell death.

Mitochondria are complex organelles that oxidize a wide range of metabolic intermediates, and their impairment has been linked to various disorders (38). Changes in the permeability and structure of the mitochondrial membrane may lead to apoptosis.

An impaired ΔΨm reflects the malfunction of mitochondria subsequent to H_2_O_2_ exposure. It also implies the decoupling of oxidative phosphorylation, accumulation of reactive oxygen species, and a reduction in cytoplasmic ATP levels. Mitochondria synthesize ATP to maintain the vital metabolism conducted in eukaryotic cells. In the early stages of cell apoptosis, the breakdown of ΔΨm regulation is one of the earliest features preceding nuclear condensation and apoptotic body formation. Data from the current study demonstrate that the ΔΨm was lower in the H_2_O_2_-treated NP cells compared with the control group, and this effect is partly abolished by CMCS in a dose-dependent manner. This indicates that the inhibitory effect of CMCS on NP cell apoptosis is associated with its protection of mitochondrial function. CMCS may protect mitochondrial function through inhibiting the reduction of ΔΨm, and thus, promote the synthesis of ATP, inhibited by H_2_O_2_ exposure.

In agreement with previous studies, the current study identified a restorative effect of CMCS on H_2_O_2_-induced ECM reduction in disc cells *in vitro*. The anti-apoptotic (anticatabolic) effects of CMCS and its potential to enhance ECM production (anabolic effect) potentially make it an excellent molecular candidate to break the cycle of degenerative cytokines that lead to further progression of IVD degeneration.

In conclusion, the current study demonstrated that CMCS can protect NP cells from H_2_O_2_-induced cell apoptosis. The mechanism of CMCS in protecting NP cells from apoptosis remains unknown, but appears to be partly mediated via caspase-3 enzymatic inhibition/Bcl-2 activation, in addition to diminishing nitric oxide production and protecting mitochondrial function. These data suggest one possible mechanism of CMCS rescue in IVD degeneration, and support the therapeutic rationale for CMCS utilization in human disc degeneration.

## Figures and Tables

**Table I:** Primers used for RT-qPCR analysis of gene expression.

Gene	Primer	Sequence	Produce size (bp)
iNOS	Forward	5′-GCAGACACATACTTTATGC-3′	445
	Reverse	5′-CAATGGCTGGTACATGGGCAC-3′	
Bcl-2	Forward	5′-GCGTCAACAGGGAGATGTCA-3	225
	Reverse	5′-GGTATGCACCCAGAGTGATG-3′	
Caspase-3	Forward	5′-GGCCTGCTTTTTACCTCAGA-3′	140
	Reverse	5′-CGTTTCCGCACAGGCTGCTT-3	
Collagen-2	Forward	5′-CCCAGAACATCACCTACCAC-3	201
	Reverse	5′-GGTACTCGATGATGGTCTTG-3	
Aggrecan	Forward	5′-GATGTCCCCTGCAATTACCA-3	230
	Reverse	5′-TCTGTGCAAGTGATTCGAGG-3	
GAPDH	Forward	5′-TGTCTCCTGCGACTTCAACAG-3′	256
	Reverse	5′-GAGGCCATGTAGGCCATGAG-3′	

RT-qPCR, reverse transcription-quantitative polymerase chain reaction; bp, base pairs; iNOS, inducible nitric oxide synthase; Bcl-2, B-cell lymphoma-2.
